# Does reduced oxygen delivery cause lactic acidosis in falciparum malaria? An observational study

**DOI:** 10.1186/s12936-019-2733-y

**Published:** 2019-03-25

**Authors:** Hugh W. Kingston, Aniruddha Ghose, Voravut Rungpradubvong, M. Trent Herdman, Katherine Plewes, Haruhiko Ishioka, Stije J. Leopold, Richard J. Maude, Benjamas Intharabut, Sanjib Mohanty, Nicholas P. J. Day, Nicholas J. White, Md Amir Hossain, Nicholas M. Anstey, Arjen M. Dondorp

**Affiliations:** 1 Mahidol Oxford Tropical Medicine Research Unit, Faculty of Tropical Medicine, Mahidol University, Bangkok, 10400 Thailand; 2 Global and Tropical Health Division, Menzies School of Health Research and Charles Darwin University, Darwin, NT 0909 Australia; 3 Chittagong Medical College, Chittagong, Bangladesh; 4 Centre for Tropical Medicine and Global Health, Nuffield Department of Clinical Medicine, Churchill Hospital, Oxford, OX3 7LJ UK; 5 Department of Clinical Tropical Medicine, Faculty of Tropical Medicine, Mahidol University, Bangkok, 10400 Thailand; 6 Ispat General Hospital, Rourkela, Orissa India; 7 Division of Cardiology, Department of Medicine, Faculty of Medicine, Chulalongkorn University, Bangkok, Thailand; 8 Cardiac Center, King Chulalongkorn Memorial Hospital, Bangkok, Thailand

**Keywords:** Malaria, Acidosis, lactic, Microcirculation, Oxygen consumption, Haemodynamics, Cardiac output

## Abstract

**Background:**

Lactic acidosis with an elevated lactate–pyruvate ratio suggesting anoxia is a common feature of severe falciparum malaria. High lactate levels are associated with parasitized erythrocyte sequestration in the microcirculation. To assess if there is an additional contribution to hyperlactataemia from relatively inadequate total oxygen delivery, oxygen consumption and delivery were investigated in patients with malaria.

**Methods:**

Adult Bangladeshi and Indian patients with uncomplicated (N = 50) or severe (N = 46) falciparum malaria or suspected bacterial sepsis (N = 27) and healthy participants as controls (N = 26) were recruited at Chittagong Medical College Hospital, Chittagong, Bangladesh and Ispat General Hospital, Rourkela, India. Oxygen delivery (DO_2_I) was estimated from pulse oximetry, echocardiographic estimates of cardiac index and haematocrit. Oxygen consumption (VO_2_I) was estimated by expired gas collection.

**Results:**

VO_2_I was elevated in uncomplicated median (IQR) 185.1 ml/min/m^2^ (135–215.9) and severe malaria 192 ml/min/m^2^ (140.7–227.9) relative to healthy persons 107.9 ml/min/m^2^ (69.9–138.1) (both p < 0.001). Median DO_2_I was similar in uncomplicated 515 ml/min/m^2^ (432–612) and severe 487 ml/min/m^2^ (382–601) malaria and healthy persons 503 ml/min/m^2^ (447–517) (p = 0.27 and 0.89, respectively). The VO_2_/DO_2_ ratio was, therefore, increased by similar amounts in both uncomplicated 0.35 (0.28–0.44) and severe malaria 0.38 (0.29–0.48) relative to healthy participants 0.23 (0.17–0.28) (both p < 0.001). VO_2_I, DO_2_I and VO_2_/DO_2_ did not correlate with plasma lactate concentrations in severe malaria.

**Conclusions:**

Reduced total oxygen delivery is not a major contributor to lactic acidosis in severe falciparum malaria.

**Electronic supplementary material:**

The online version of this article (10.1186/s12936-019-2733-y) contains supplementary material, which is available to authorized users.

## Background

Severe falciparum malaria is a life-threatening infection requiring prompt treatment with intravenous artesunate [1]. Patients present with a range of syndromes reflecting vital organ dysfunction. The depth of coma, degree of metabolic/lactic acidosis and severity of renal impairment are the major prognosticators for a fatal outcome. During blood stage infection with *Plasmodium falciparum,* 48-h cycles of asexual replication in red cells result in exponential growth of the infecting parasite biomass and the corresponding release of haemoglobin and haemozoin pigment-containing digestive vacuoles at schizont rupture. As the *P. falciparum* parasites mature the infected red cells sequester heterogeneously in the microvasculature causing microvascular obstruction and impairment of tissue perfusion [1]. Plasma lactate is elevated in relation to the proportion of non-perfused capillaries [2]. Hyperlactataemia is associated with increased glucose turnover [3] and increased lactate-pyruvate ratios [4], consistent with hypoxia rather than hypermetabolism as the cause. Increased plasma lactate also correlates inversely with functional liver blood flow suggesting impaired hepatic clearance contributes to hyperlactataemia [5].

Previous studies have investigated the haemodynamics of severe malaria using invasive techniques [2, 6–8] and reported lack of association between total oxygen delivery and plasma lactate [2]. In this article the hypothesis that inadequate oxygen delivery contributes to the metabolic acidosis of severe malaria is evaluated. Oxygen consumption was measured to assess whether inhibition of oxidative metabolism contributes to lactic acidosis, and determined the ratio of oxygen consumption to delivery (VO_2_/DO_2_) to provide a better estimate of the adequacy of oxygen delivery.

## Methods

### Patients

Adult patients with severe or uncomplicated falciparum malaria, patients with suspected bacterial sepsis of any severity meeting the systemic inflammatory response (SIRS) criteria, and healthy adult volunteers were recruited in Chittagong Medical College Hospital (CMCH), Chittagong, Bangladesh and Ispat General Hospital (IGH), Rourkela, India. Patients with malaria had asexual stages visible on a thick or thin blood film. Severe malaria was defined as being present when one or more of the following features were present: cerebral malaria (Glasgow coma score < 11); parasites > 100,000/mm^3^ with either severe anaemia (haematocrit < 20%) or bilirubin level of > 2.5 mg/dl; renal failure (creatinine > 265 μmol/l or anuria); hypoglycaemia < 2.2 mmol/l; systolic blood pressure < 80 mmHg with cold extremities; pulmonary oedema; spontaneous bleeding; generalized convulsions (> 1 in 24 h); venous bicarbonate < 15 mmol/l, hyperparasitaemia > 10%, venous lactate > 4 mmol/l. Participants with paired oxygen consumption and oxygen delivery estimates were included in this analysis. All participants (or legally acceptable representatives in cases where patients lacked capacity during the acute illness) gave informed, written consent. The study was approved by the Oxford Tropical Research, Chittagong Medical College and IGH ethical committees. Patients with severe malaria received intravenous artesunate, and those with uncomplicated malaria received either oral artemether–lumefantrine (Chittagong) or artesunate–sulfadoxine/pyrimethamine (Rourkela) according to national guidelines.

On enrolment a history and examination were recorded, and blood for biochemistry and haematology was collected. Plasma lactate and base excess were measured by iSTAT (Abbott laboratories, Illinois, USA). Plasma *Plasmodium falciparum* histidine rich protein 2 (PfHRP2) was measured by ELISA (Cellabs, Brookvale, Australia). Plasma cell free haemoglobin was measured by ELISA (Bethyl laboratories, Texas, USA). Plasma asymmetric dimethyl arginine (ADMA) and arginine were estimated by HPLC. Acute kidney injury (AKI) was staged based on enrolment plasma creatinine and baseline creatinine estimated by back-calculation using the Modification of Diet in Renal Disease equation [9]. This assumed a baseline glomerular filtration rate of 75 ml/min. Stage 1 AKI was defined as creatinine 1.5–1.9× baseline, stage 2 as creatinine 2–2.9× baseline, and stage 3 as creatinine ≥ 3× baseline or creatinine 353.6 µmol/l. The coma acidosis malaria (CAM) score was calculated using Glasgow coma score and base excess as previously [10].

### Oxygen consumption and delivery

A timed collection of expired air (typically 45 s) was collected from participants into a Douglas bag using a mask and three way tap with one-way valves [11]. When conscious, participants were asked to breathe normally. The measurement was not possible in patients requiring supplementary oxygen or nasogastric tubes. The volume of gas (RSS 100HR Research Pneumotach, Hans Rudolph, Shawnee, Kansas, USA) and partial pressures of oxygen (Model MO-200, Apogee instruments, Logan, Utah, USA) and carbon dioxide (Tidal Wave S Capnograph, Novametrix Medical Systems, Wallingford, CT, USA) in the bag were measured and used to calculate oxygen consumption (VO_2_) and carbon dioxide production (VCO_2_) [11]. The respiratory quotient (RQ) was calculated as the ratio of VCO_2_/VO_2_. VO_2_ and VCO_2_ were indexed to body surface area (Haycock) (VO_2_I, VCO_2_I, respectively). The within participant standard deviations for VO_2_I, VCO_2_I and RQ estimated in 11 patients with malaria were 36 ml/m^2^, 30 ml/m^2^ and 0.15, respectively. Cardiac output was estimated by transthoracic echocardiography using heart rate, aortic velocity time integral and left ventricular outflow tract diameter [12]. Arterial blood oxygen content was calculated from haemoglobin and oxygen saturation (by pulse oximetry) ignoring dissolved oxygen [13]. Oxygen delivery (DO_2_) was calculated as the product of arterial blood oxygen content and cardiac output and indexed to body surface area (DO_2_I) [13]. The ratio of VO_2_/DO_2_ was then calculated.

### Statistics

Data were analysed using Stata version 14 (StataCorp, Texas, USA). Correlations were assessed using Spearman’s rank. Kruskal–Wallis tests or chi^2^ tests were used to compare continuous and binary data across groups.

## Results

### Patients and outcomes

Baseline characteristics and outcome in the different groups are shown in Table 1. A total of 97 patients were studied in Bangladesh and 26 in India. Mortality was 11/46 (24%) in severe malaria and 5/27 (19%) in the sepsis groups. In severe malaria the median CAM score was 2 (IQR 2 to 3).

**Table 1:** Participant characteristics

Variable	Healthy (N = 26)	Uncomplicated malaria (N = 50)	Severe malaria (N = 46)	Sepsis (N = 27)	*p* value
Sex (% male)	81	78	67	56	0.12
Coma (% GCS < 11)	0	0	57	15	< 0.001
Lactate > 4 mmol/l (%)	0	0	46	4	< 0.001
Creatinine > 3 mg/dl (%)	0	0	26	7	< 0.001
Died (%)	0	0	24	19	0.001
Age (years)	28 (26 to 35)	25 (20 to 40)	35 (26 to 42)	32 (23 to 55)	0.196
Temperature (°C)	36.8 (36.6 to 37)	37.5 (36.9 to 38.7)	37.6 (37.1 to 38.7)	38.8 (38.2 to 39.3)	< 0.001
Heart rate (/min)	67 (60 to 74)	95 (85 to 112)	105 (87 to 123)	102 (88 to 114)	< 0.001
Base excess (mM)	2 (− 1 to 2)	− 1 (− 3 to 1)	− 7 (− 11 to − 2)	1 (− 3 to 3)	< 0.001
Plasma lactate (mM)	1.1 (0.9 to 1.4)	1.5 (1.1 to 2)	3.7 (2.5 to 6.5)	1.7 (1.1 to 2.2)	< 0.001
Creatinine (mg/dl)	0.9 (0.8 to 1)	1 (0.8 to 1.2)	1.4 (1 to 3.3)	1 (0.9 to 1.3)	< 0.001
Parasitaemia (/μl)	NA	18,903 (3768 to 56,796)	74,920 (19,091 to 282,751)	NA	0.002
Plasma *Pf*HRP2 (ng/ml)	NA	312 (172 to 763)	2492 (1664 to 3950)	NA	< 0.001
Plasma arginine (μM)	101 (85 to 107)	52 (37 to 64)	53 (37 to 75)	49 (39 to 54)	< 0.001
Plasma ADMA (μM)	0.53 (0.48 to 0.56)	0.58 (0.48 to 0.75)	0.58 (0.47 to 0.89)	0.5 (0.39 to 0.61)	0.032
Plasma arginine/ADMA	186 (160 to 227)	87 (70 to 112)	77 (66 to 98)	92 (68 to 132)	< 0.001
Plasma CFH (μM)	2.7 (1.3 to 4.8)	3.6 (1.9 to 5.1)	8 (3.8 to 14.5)	2.3 (0.6 to 5.6)	< 0.001
VO_2_I (ml/min/m^2^)	107.9 (69.9 to 138.1)	185.1 (135 to 215.9)	192 (140.7 to 227.9)	155.3 (132.1 to 196.4)	< 0.001
VCO_2_I (ml/min/m^2^)	86.3 (56.5 to 105.6)	118.3 (89.3 to 155)	132.6 (98.4 to 145.6)	105.2 (73.9 to 131.3)	0.003
RQ	0.77 (0.7 to 0.86)	0.63 (0.58 to 0.76)	0.67 (0.59 to 0.74)	0.64 (0.59 to 0.83)	0.004
CI (ml/min/m^2^)	2575 (2340 to 3111)	3792 (3404 to 4439)	4167 (3564 to 4876)	4142 (2988 to 4916)	< 0.001
Haematocrit (%)	43 (38 to 46)	32 (25 to 37)	26 (20 to 35)	37 (30 to 42)	< 0.001
O_2_ saturation (%)	97 (96 to 98)	97 (96 to 99)	96 (95 to 97)	95 (92 to 97)	< 0.001
DO_2_I (ml/min/m^2^)	503 (447 to 517)	515 (432 to 612)	487 (382 to 601)	575 (513 to 694)	0.02
VO_2_/DO_2_	0.23 (0.17 to 0.28)	0.35 (0.28 to 0.44)	0.38 (0.29 to 0.48)	0.26 (0.21 to 0.34)	< 0.001

For non-binary variables statistics shown are median (interquartile range)

*VO*_*2*_*I* oxygen consumption index, *VCO*_*2*_*I* carbon dioxide production index, *RQ* respiratory quotient, *CI* cardiac index, *DO*_*2*_*I* oxygen delivery index, *NA* not applicable. *CFH* cell free haemoglobin

P-value is for Kruskal–Wallis test across the four groups or chi^2^ test

### VO_2_I, VCO_2_I, DO_2_I and their ratios in the different patient groups

**Fig. 1:**
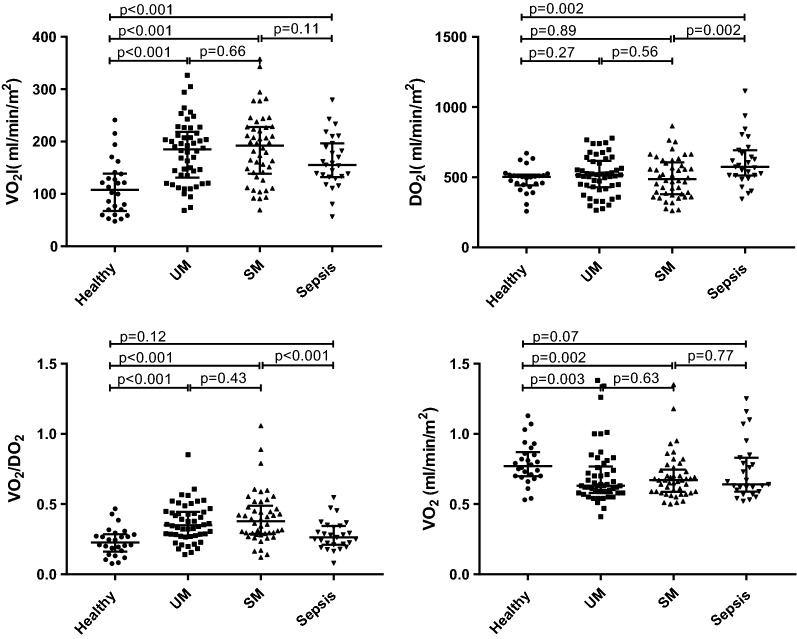
Oxygen consumption and delivery indices in different groups of participant. UM, uncomplicated falciparum malaria; SM, severe falciparum malaria. Bars indicate medians and interquartile ranges. VO_2_I, oxygen consumption index; DO_2_I, oxygen delivery index; VO_2_I/DO_2_I, ratio of oxygen consumption to delivery; RQ, respiratory quotient

Compared to healthy participants, oxygen consumption (Fig. 1, Table 1) was higher in uncomplicated and severe malaria and sepsis (all p < 0.001). Oxygen consumption was not different between patients with uncomplicated versus severe falciparum malaria (p = 0.655) or sepsis versus severe falciparum malaria (p = 0.105). VCO_2_I was significantly higher in uncomplicated and severe malaria and sepsis than in healthy participants (p = 0.002, p < 0.001, p = 0.022 respectively). Despite the increased VO_2_I in malaria, oxygen delivery (Table 1) was similar in both uncomplicated and severe malaria to values in healthy persons (p = 0.27 and 0.89, respectively), but was significantly higher in sepsis than health (p = 0.002). Consequently, VO_2_/DO_2_ values were higher in both uncomplicated and severe malaria than in healthy persons (both p < 0.001) or sepsis (p = 0.005 and p < 0.001, respectively), but were similar between patients with sepsis and healthy participants (p = 0.117). There was no difference in the VO_2_/DO_2_ ratio between uncomplicated and severe malaria patients (p = 0.433). Compared to healthy subjects the respiratory quotient was reduced in uncomplicated and severe malaria but not sepsis (p = 0.003, 0.002, 0.072, respectively). Similar results were found for VO_2_, DO_2_ and VO_2_/DO_2_ when the subset of severe malaria patients with hyperlactataemia were compared to the control groups (Additional file 1).

### VO_2_I, VCO_2_I, DO_2_I and their ratios in severe malaria and sepsis

**Fig. 2:**
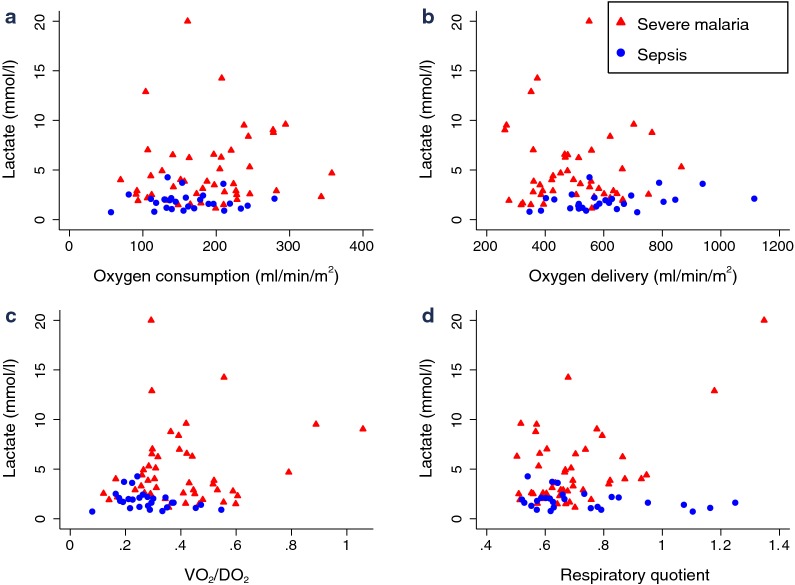
Oxygen consumption and delivery indices and plasma lactate in patients with severe malaria or sepsis.** a**: Lactate and oxygen consumption.** b**: Lactate and oxygen delivery.** c**: Lactate and VO_2_/DO_2_.** d**: Lactate and respiratory quotient. VO_2_/DO_2_, ratio of oxygen consumption to delivery

In severe malaria VO_2_I, DO_2_I and VO_2_I/DO_2_I were not significantly different in patients with or without hyperlactataemia (all p > 0.05). There was no correlation between VO_2_I, DO_2_I, RQ or the ratio of VO_2_/DO_2_ and plasma lactate in severe malaria or sepsis (all p > 0.05, Fig. 2). There was however a positive correlation between VCO_2_ and plasma lactate in severe malaria (ρ = 0.34, p = 0.02, N = 46). Plasma lactate correlated with parasitaemia (ρ = 0.40, p = 0.006, N = 46) and plasma PfHRP2 (ρ = 0.30, p = 0.04, N = 46) as markers of parasite biomass. In severe malaria, base excess correlated positively with DO_2_I (ρ = 0.29, p = 0.049, N = 46), negatively with VO_2_/DO_2_ (ρ = − 0.30, N = 46, p = 0.04) but not RQ, VO2_2_I or VCO_2_I. In a multivariate linear regression model for admission base excess in severe malaria patients using AKI (categorical) and VO_2_/DO_2_ or DO_2_I as independent variables, AKI but not VO_2_I/DO_2_ or DO_2_I remained a significant predictor of base excess in the model.

Oxygen consumption did not correlate with markers of nitric oxide bioavailability in severe malaria (plasma arginine, asymmetric dimethylarginine (ADMA), the arginine/ADMA ratio, plasma cell free haemoglobin) or with temperature (p > 0.05). There was no correlation between oxygen consumption and measures of parasite biomass (plasma *Pf*HRP2 and parasitaemia) (p > 0.05).

## Discussion

Severe and uncomplicated falciparum malaria were both accompanied by an approximate 75% increase in VO_2_I compared to healthy individuals. The increase in VCO_2_I was approximately 50%. In patients admitted with suspected bacterial sepsis these values were also higher than in healthy subjects but the increments were approximately half those observed in patients with malaria. Cardiac index was increased in malaria, but unlike sepsis this did not result in a rise in DO_2_I due to anaemia. Consequently, the ratio of oxygen consumption to delivery rose in both severe and uncomplicated malaria but not sepsis. VO_2_/DO_2_ and DO_2_I did not correlate with lactate in severe malaria or in sepsis consistent with tissue hypoxia not being determined by overall oxygen delivery.

The increase in VO_2_I and VCO_2_I observed in both severe and uncomplicated malaria and sepsis is consistent with an increase in metabolic rate. The increase observed in severe malaria is consistent with a previous small study using invasive techniques [14]. A previous study in sepsis found elevated VO_2_ in sepsis, which decreased as disease severity increased [15]. This inverse relationship with severity was not found in malaria. The lack of an inverse relationship with plasma lactate indicates that widespread mitochondrial dysfunction, caused by hypoxia or other factors, is not a major contributor to acidosis in severe malaria. In conditions with mitochondrial dysfunction, such as biguanide intoxication, a low VO_2_ and inverse relationship with lactate is observed [16]. From the perspective of increases in VO_2_ on exercise, the increase in severe malaria of about 1.8-fold appears modest relative to the tenfold increase from rest expected when running at 6 miles per hour [17].

Tissue oxygen uptake may be limited by diffusion or convection [18]. In severe falciparum malaria, microvascular obstruction resulting from sequestration of erythrocytes containing mature parasites has been directly visualized [19] and results in focal tissue hypoxia [1]. The VO_2_/DO_2_ ratio, which is numerically equivalent to the oxygen extraction ratio [13] was elevated in both severe and uncomplicated malaria. Despite an increase in VO_2_/DO_2_ in malaria, which would be expected to result in a fall in end-capillary oxygen tension, no relationship between VO_2_/DO_2_ and plasma lactate was observed. Consistent with previous findings, no relationship between DO_2_ and plasma lactate was found [2]. The lack of relationship between VO_2_I/DO_2_ or DO_2_ and plasma lactate suggests that DO_2_ is adequate and that variation in the oxygen content of perfused capillaries does not increase oxygen tension significantly around non-perfused capillaries. This could be because non-perfused capillaries occur in small patches as seen in malaria retinopathy observed by fluorescein angiography [20] or because of microvascular dysfunction in perfused capillaries with failure of compensatory responses [21].

The reason for the elevated VO_2_I in both severe and uncomplicated malaria is likely multifactorial. Hypermetabolism from the host inflammatory response and increased cardiac work and work of breathing may contribute to the elevation in VO_2_I. Elevated catecholamines in malaria and sepsis may increase the VO_2_ of many organs including skeletal muscle [22], possibly due in part to mitochondrial uncoupling. Nitric oxide can inhibit mitochondrial respiration and reduced NO bioavailability in malaria could therefore result in an increased VO_2_I [21]; however, no association between markers of NO bioavailability and VO_2_I was observed. While whole body oxygen consumption is increased in malaria, this increase is probably heterogeneous across different tissues. Previous studies have investigated oxygen consumption in different organs. In cerebral malaria, despite normal blood flow cerebral oxygen consumption was reduced and correlated inversely with lactate production, consistent with hypoxia driving anaerobic glycolysis [7]. Skeletal muscle oxygen consumption assessed with near infrared spectroscopy (NIRS) was found to be higher in malaria than healthy individuals, but similar in severe and uncomplicated malaria [21]. In severe malaria, an inverse relationship between muscle oxygen consumption and plasma lactate was noted, consistent with inadequate oxygen availability resulting in anaerobic glycolysis [21]. Whilst anaerobic glycolysis does not consume oxygen directly, clearance of the resulting lactate via gluconeogenesis (Cori cycle) or oxidation does. As such, certain organs (the brain) or small hypoxic patches of tissue may have a reduced VO_2_ and produce lactate which may be cleared in other areas which have an increased VO_2_. Patients with severe malaria have increased metabolic requirements as evidenced by their increased VO_2_.

This study had several shortcomings: the number of patients with the different syndromes of severe malaria and sepsis was relatively small. There were too few septic patients to determine the relationship between VO_2_ and severity in this category. The assessment of oxygen consumption and CO_2_ production from expired gas assumes steady state conditions; application of the mask may have caused hyperventilation, increasing the respiratory minute volume and hence CO_2_ elimination in particular.

Inadequate DO_2_I is unlikely to contribute to tissue hypoxia in severe malaria. Future studies should examine the potential role of blood transfusion and fluid therapy in severe malaria in preserving haemodynamic stability and renal function as opposed to improving lactic acidosis. Whilst blood transfusion is unlikely, except in extreme anaemia, to improve outcome due to reducing tissue hypoxia, it might lessen the chance of cardiovascular decompensation developing by increasing the cardiac index reserve. The optimal blood transfusion threshold in adult severe malaria has not been well established. A large randomized controlled trial of blood transfusion in paediatric severe malaria is ongoing (ISRCTN84086586).

## Conclusion

Falciparum malaria was associated with increased oxygen consumption but this was not related to disease severity. Lactic acidosis did not result from inadequate overall macrocirculatory tissue oxygen delivery, but more likely from patchy microvascular perfusion abnormalities combined with impaired hepatic clearance. How the haemodynamic status can be optimized to avoid decompensation in the period after antimalarial treatment needs further investigation.

## Additional file

**Additional file 1: Table S1.** Comparison of hyperlactatemic severe malaria patients with control groups.

## Abbreviations

ADMA: asymmetric dimethyl arginine
AKI: acute kidney injury
CAM: coma acidosis malaria
CI: cardiac index
CFH: cell free haemoglobin
CMCH: Chittagong Medical College Hospital
DO_2_: oxygen delivery
DO_2_I: oxygen delivery index
IGH: Ispat General Hospital
IQR: interquartile range
PfHRP2: *Plasmodium falciparum* histidine rich protein 2
RQ: respiratory quotient
SM: severe falciparum malaria
UM: uncomplicated falciparum malaria
VCO_2_: carbon dioxide production
VCO_2_I: carbon dioxide production index
VO_2_: oxygen consumption
VO_2_I: oxygen consumption index
VO_2_/DO_2_: ratio of oxygen consumption to delivery
