# Supplementary material for: Does reduced oxygen delivery cause lactic acidosis in falciparum malaria? An observational study
Source: Malar J. 2019 Mar 25;18:97. doi: 10.1186/s12936-019-2733-y (PMC6434797; doi:10.1186/s12936-019-2733-y)
Supplement: Supplementary file 1 — Additional file 1: Table S1. Comparison of hyperlactatemic severe malaria patients with control groups. [file 12936_2019_2733_MOESM1_ESM.docx]

|  |  |  |  |  |  | p-values | | | |
| --- | --- | --- | --- | --- | --- | --- | --- | --- | --- |
|  | Healthy (HC) | Uncomplicated malaria (UM) | Severe malaria (SM) - lactate >4mmol/l | Sepsis (SE) |  | Overall | HC vs SM (lactate>4) | UM vs SM (lactate>4) | SE vs SM (lactate>4) |
| N | 26 | 50 | 21 | 27 |  |  |  |  |  |
| VO_2_I (ml/min/m2) | 108 (70 to 138) | 185 (135 to 216) | 205 (141 to 244) | 155 (132 to 196) |  | <0.001 | <0.001 | 0.427 | 0.216 |
| VCO_2_I (ml/min/m2) | 86 (56 to 106) | 118 (89 to 155) | 141 (105 to 158) | 105 (74 to 131) |  | 0.001 | <0.001 | 0.162 | 0.387 |
| RQ | 0.77 (0.7 to 0.86) | 0.63 (0.58 to 0.76) | 0.69 (0.58 to 0.86) | 0.64 (0.59 to 0.83) |  | 0.038 | 0.178 | 0.296 | 0.501 |
| CI (ml/min/m2) | 2575 (2340 to 3111) | 3792 (3404 to 4439) | 4367 (3801 to 4876) | 4142 (2988 to 4916) |  | <0.001 | <0.001 | 0.047 | 0.522 |
| Hematocrit (%) | 43 (38 to 46) | 32 (25 to 37) | 23 (20 to 34) | 37 (30 to 42) |  | <0.001 | <0.001 | 0.052 | 0.005 |
| O_2_ saturation (%) | 97 (96 to 98) | 97 (96 to 99) | 96 (95 to 97) | 95 (92 to 97) |  | 0.001 | 0.142 | 0.153 | <0.001 |
| DO_2_I (ml/min/m2) | 503 (447 to 517) | 515 (432 to 612) | 476 (422 to 558) | 575 (513 to 694) |  | 0.014 | 0.898 | 0.504 | 0.021 |
| VO_2_/DO_2_ | 0.23 (0.17 to 0.28) | 0.35 (0.28 to 0.44) | 0.32 (0.3 to 0.42) | 0.26 (0.21 to 0.34) |  | <0.001 | <0.001 | 0.753 | 0.005 |

**Table S1. Comparison of hyperlactatemic severe malaria patients with control groups**

Statistics shown are median (interquartile range). VO_2_I = oxygen consumption index, VCO_2_I = carbon dioxide production index, RQ = respiratory quotient, CI = cardiac index, DO_2_I = oxygen delivery index. NA = not applicable. Overall p-value is for Kruskal-Wallis test across the four groups, Mann-Whitney U-test for comparisons between two groups.
